# Mindfulness-based interventions and their relationships with body image and eating behavior in adolescents: a scoping review

**DOI:** 10.1186/s40337-025-01238-6

**Published:** 2025-05-01

**Authors:** Silvia Patrícia de Oliveira Silva Bacalhau, Luciana Gonçalves de Orange, Marco Aurélio de Valois Correia Junior, Juliana Vieira Nunes, Carolina Larissa Alves Sales de Almeida, Maria Wanderleya de Lavor Coriolano-Marinus

**Affiliations:** 1 Graduate Program in Child and Adolescent Health, Federal University of Pernambuco, Av. Prof. Luiz Freire, 500 - University City, Recife, 50740-545 PE Brazil; 2 University of Pernambuco, Recife, PE Brazil; 3 Vitoria Academic Center , Federal University of Pernambuco, Vitória de Santo Antão, PE,, Brazil; 4 Federal Institute of Education, Science and Technology of Pernambuco, Recife, PE, Brazil

**Keywords:** Adolescent, Body image, Feeding behavior, Mindfulness-based interventions, Mindful eating, Mindfulness

## Abstract

**Background:**

During adolescence, there are risks of changes in eating behavior and concerns about weight and body shape. Mindfulness-based interventions (MBIs) may bring benefits to this population.

**Objective:**

This study aimed to assess research that carried out MBIs and its repercussions onbody image and eating behavior in non-clinical adolescents.

**Methods:**

This is a scoping review study that followed the JBI methodology and the Preferred Reporting Items for Systematic reviews and Meta-Analyses extension for Scoping Reviews (PRISMA-ScR) protocol, and was registered in the Open Science Framework (10.17605/OSF.IO/3VB6K). An initial search in MEDLINE (PubMed) was performed, followed by analysis of the words contained in the titles, abstracts and indexed terms. The identified keywords and indexed terms (“adolescent”, “body image”, “feeding behavior”, “mindfulness”, “mindful eating”, “body dissatisfaction” and “body dysmorphic disorders”) were used for research in PubMed, Web of Science, Embase and Scopus. There was no language restriction and the selection considered articles published up to December 2023. Duplicate articles were removed and, after analyzing the title, abstract and keywords, the selected articles were read in full, excluding those that did not meet the proposed eligibility criteria. The references of the initially selected articles were researched in additional sources.

**Results:**

Six studies out of a total of 665 were found. Five studies were randomized controlled trials and one was a single-arm pilot study. Of the six studies, three found positive relationships between MBIs and eating behavior (increased awareness of eating behaviors, significant reductions in dietary restraint and eating disorder symptoms) and/or body image (reduced concerns about weight and body shape, as well as negative affect - through improved emotional regulation). Three studies did not find statistically significant results.

**Conclusion:**

MBIs appear to improve eating behavior and body image acceptance; however, further research is needed to draw more robust conclusions. Heterogeneity among the methodologies used was observed: variations in sample sizes, age groups and gender of participants, and protocols used, which prevents generalization of the results to adolescents and corroborates the need to create a protocol based on information already available in the literature.

**Supplementary Information:**

The online version contains supplementary material available at 10.1186/s40337-025-01238-6.

## Abstract (plain language summary)

This scoping review presents a survey of articles on mindfulness-based interventions (MBIs) applied to non-clinical adolescents and their relationships with body image and eating behavior. During adolescence, physical, physiological, psychological, social and emotional changes occur and there is an increase in emotional vulnerability. MBIs can bring benefits to this population, as they are already used in therapeutic processes, for instance. In the literature, studies involving MBIs with adolescents, eating behavior and body image are still incipient. Mapping the contexts and duration of MBIs can assist in the construction and implementation for researchers and clinicians in promoting dietary health and preventing situations such as body dissatisfaction/distortion and eating disorders. Thus, MBIs can be part of adjuvant treatments for these conditions.

**Supplementary Information:**

The online version contains supplementary material available at 10.1186/s40337-025-01238-6.

## Introduction

Adolescence is a phase of life characterized by physical, physiological, psychological and emotional changes. Exposure to thoughts that negatively influence the control of emotions can have repercussions on various aspects of daily life, including diet and physical fitness [1, 2].

Eating behavior formation begins in the prenatal period and is consolidated in adult life [3]. During adolescence, this behavior is influenced positively or negatively by factors such as practicing restrictive diets, sharing family meals, conversations and teasing about weight among family members and other people important to adolescents, and self-assessment of body image [4, 5].

Disordered eating behavior or even an eating disorder can arise from environmental influences and lead individuals to an inadequate relationship with food and their body [6, 7]. Sociocultural factors such as family, peers and the media play a role in the formation of adolescents’ body image, according to the Tripartite Influence Model of Thompson et al. [8].

Body image is the perspective that individuals have of their body: how they imagine themselves, see themselves, feel, perceive themselves [5]. Their dissatisfaction generates consequences such as social, academic, professional, relationship, identity and cognitive losses, in addition to being associated with depreciation of sleep quality and a decrease in psychological well-being [9]. Body dissatisfaction makes it difficult to engage in self-care behaviors among young people, resulting in a risk for engaging in disordered eating behaviors [10–12].

Despite the influence of sociocultural factors on body image formation, it is important to mention that some of these can also act as protective factors. Family, a positive school environment, and support from teachers can provide tools to promote self-esteem, security, and confidence, contributing to strategies for dealing with the impact of social media messages [13].

Health promotion programs for adolescents with the aim of improving body image can prevent problems related to eating and weight, and universal programs applied in schools collaborate with prevention and/or identification of adolescents with subclinical or already established eating disorders [14].

Mindfulness-based interventions (MBIs) may be promising with adolescents given their purpose of promoting greater awareness of the present moment, reducing automatic responses and helping to improve self-knowledge, self-compassion and non-judgment [15]. MBIs can reduce concerns about weight/shape, in addition to acting on dietary restriction, reducing the Body Mass Index (BMI), eating in the absence of hunger, binge eating, increased willingness to eat new healthy foods and reduced psychopathology of eating disorders [14, 16].

There is little research in the literature on MBIs, body image and eating behavior among adolescents (especially in universal prevention settings, such as schools). There is a need for intervention studies that are appropriate for this population, with appropriate designs, that assess the effect of the proposed intervention. In existing literature, there are questions about the most appropriate age to work on the topics, content and duration, in addition to clear evidence on acceptability and efficacy [6, 17]. The variability in study designs also indicates the need for more research with controlled conditions [16]. Based on these gaps, this scoping review aims to bring to light current contributions to the advancement of knowledge in this field of study.

A preliminary search in PROSPERO, MEDLINE, the Cochrane Database of Systematic Reviews and the JBI Evidence Synthesis showed that no current or ongoing scoping or systematic reviews on the topic were found.

This review aimed to assess research that carried out MIBs and its repercussions on eating behavior and body image in non-clinical adolescents. The following research question was formulated: what is the evidence on the effects of MBIs on body image and/or eating behavior in non-clinical adolescents?

## Methods

This scoping review was conducted following JBI methodology for scoping reviews [18] and using Preferred Reporting Items for Systematic reviews and Meta-Analyses extension for Scoping Reviews (PRISMA-ScR) [19]. The protocol was registered in the Open Science Framework (OSF) with the link: 10.17605/OSF.IO/3VB6K.

### Eligibility criteria

All types of articles published until December 2023 in peer-reviewed scientific journals and written without language restrictions were included. Information such as the MBI protocol used, duration of intervention and duration of each session, presence and time of follow-up application, which guided practice, main results and conclusions were identified.

Reviews, book chapters, abstracts, preprints, theses and articles focusing on therapies/treatments were excluded. Studies whose population had any associated condition, pathology or procedures were also excluded.

### Research strategy

A three-stage search strategy was used for this review. An initial search in MEDLINE (PubMed) was carried out, followed by analysis of the words contained in the titles, abstracts and indexed terms used in articles.

A second search using identified keywords and indexed terms (“adolescent”, “body image”, “feeding behavior”, “mindfulness” and “mindful eating”, “body dissatisfaction” and “body dysmorphic disorders”) was carried out in the chosen databases. Soon after, the third stage searched for additional sources for the list of references of articles initially selected.

Charts 1 and 2 present search strategies carried out on February 16, 2022 and updated on December 5, 2023, using the “advanced search” feature with MeSH (Medical Subject Headings) descriptors and controlled vocabulary developed by the U.S. National Library of Medicine and the term “AND” as a Boolean operator.

**Chart 1:** Search strategy using Web of Science, Embase, Scopus and PubMed databases

Descriptors and crossings
1. Adolescent AND Mindfulness AND Feeding behavior
2. Adolescent AND Mindfulness AND Body image
3. Adolescent AND Mindful eating AND Feeding behavior
4. Adolescent AND Mindful eating AND Body image
5. Adolescent AND Mindfulness AND Body dissatisfaction
6. Adolescent AND Mindfulness AND Body dysmorphic disorders
7. Adolescent AND Mindful eating AND Body dissatisfaction
8. Adolescent AND Mindful eating AND Body dysmorphic disorders

The Population, Concept and Context (PCC) strategy was used to guide the searches:

**Chart 2:** PCC strategy used to guide research

**P**opulation: non-clinical adolescent human subjects aged 10 to 18 years;
**C**oncept: body image and/or eating behavior;
**C**ontext: evidence on the effects of Mindfulness-Based Interventions.

### Selection of sources of evidence

This scoping review considered experimental and quasi-experimental studies, including randomized and non-randomized clinical trials, before-and-after studies, and time series. Observational studies, including cohort studies, case-control studies, and cross-sectional studies, were also considered. This review did not consider series and case reports, protocols, study designs and clinical practice guidelines, literature reviews, conference abstracts, books/book chapters, dissertations/theses and text and opinion articles.

**Fig. 1:**
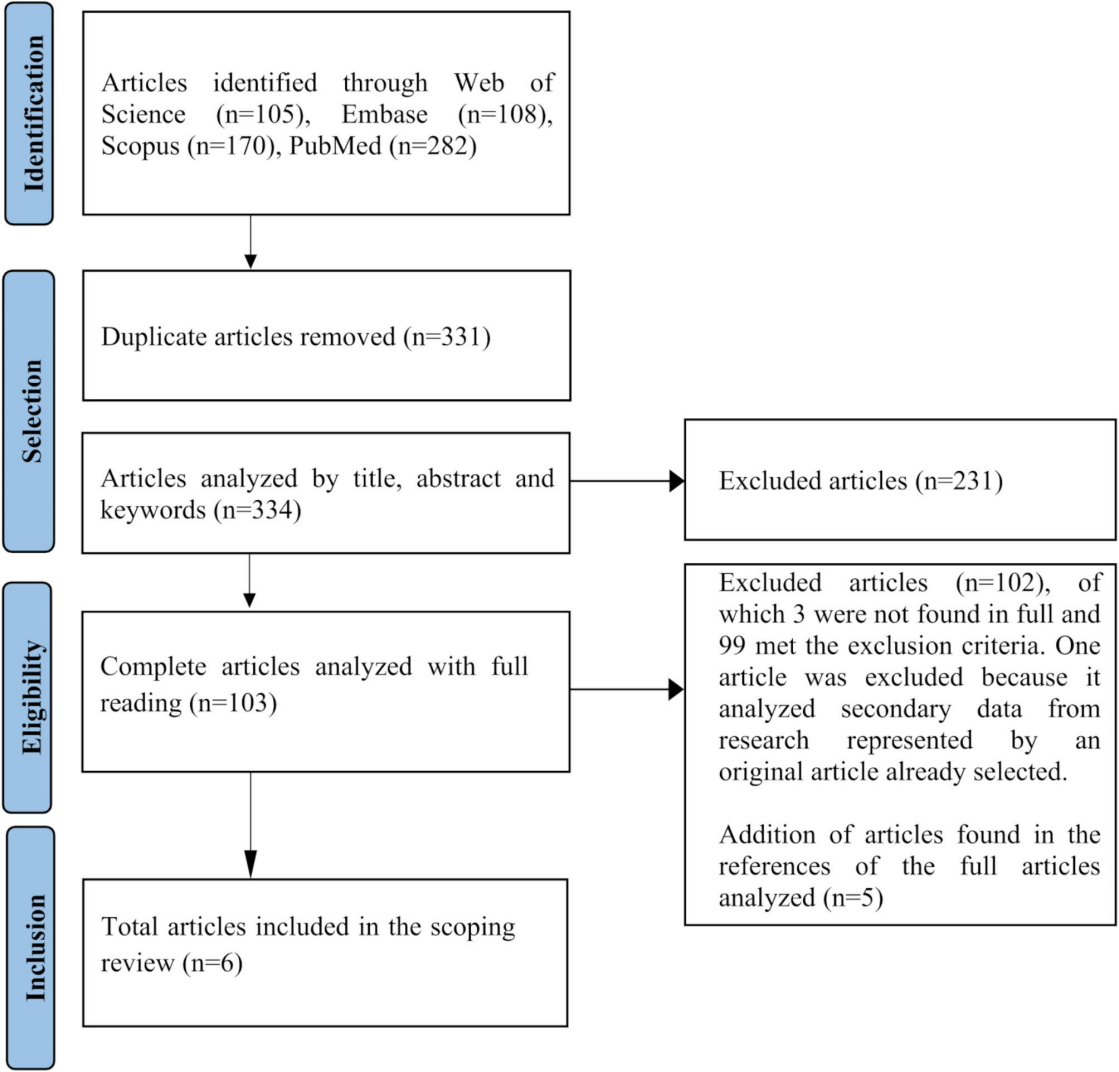
Flowchart adapted from PRISMA-ScR, developed by Moher et al. (2009), representing the scoping review process

Databases searched included MEDLINE (PubMed), Scopus, Embase, and Web of Science. The research was limited to humans. There was no language limitation and articles with a publication date up to December 5, 2023 were selected. Figure 1 shows the flow of the study selection process, drawn up based on PRISMAScR recommendations, representing the process of identification and the number of records added or removed at each stage.

Article selection was carried out by three reviewers (SPOSB; CLASA; JVN), who examined each record independently. Any disagreements were resolved by a fourth researcher and resolutions were reached by consensus among the authors in online meetings.

The research results were managed using the Mendeley reference manager (mendeley.com).

### Data extraction

Three researchers (SPOSB; MWLCM; LGO) developed a spreadsheet to extract relevant data from each of the six included studies. For each study, author Bacalhau, SPOS extracted data according to authors, year of publication, country of study, study design, sample size and sex, age of participants, objectives, interventions such as frequency, duration, presence of follow-up up, instructor, main results and conclusions. Chart 3 presents the final version of a spreadsheet with the main characteristics of studies included in the review [See Additional File 1].

## Results

### Selection of sources of evidence

A total of 665 database records were downloaded; of these, 331 were duplicates, leaving 334 studies to assess relevance, initially by reading titles and abstracts, of which 231 were excluded. There were 103 articles left to read in full texts, which were added to five relevant articles found through cross-referencing. Of this total of 108 articles, 102 were excluded after reading the full text. The final review sample consisted of six studies.

Of the six studies selected, three studies took place in Australia [6, 14, 17, 20], two in the United States [21, 22] and one in Germany [23]. Five recruited participants in a school setting [6, 14, 17, 20, 22, 23] and one through flyers distributed and posted in community organizations and online [21].

All selected studies were published in the last nine years.

Regarding the profile of the populations investigated, one study worked only with females [14, 20], and the others, with adolescents of both sexes. Regarding age groups, four of studies analyzed populations aged between 12 and 15 years [6, 17, 22, 23], and in the remaining two, ages were between 14 and 18 years old [14, 20, 21].

In relation to race/ethnicity, two studies cited a white predominance (60 and 93%, respectively) [21, 22]; one study had the majority of the population cited as Caucasian (82.9 and 84%) [14, 20]; and three studies did not mention race/ethnicity [6, 17, 23].

### Samples and data collection

There was a wide range of sample sizes, with the smallest recorded sample of 15 participants [21] and the largest of 1,654 participants [23]. Three studies considered, in addition to quantitative data, qualitative data [14, 17, 23].

The data collection instruments used to examine mindfulness status, eating behavior and body image varied between studies. The main ones were the Child and Adolescent Mindfulness Measure (CAMM) (Greco et al., 2011) and Comprehensive Inventory of Mindfulness Experience - Adolescents (CHIME-A) (Johnson, C., et al. 2017), used to examine mindfulness status. To analyze eating behavior, we used Eating Disorder Examination Questionnaire (EDE-Q) (Fairburn & Beglin, 1994), Eating Disorder Inventory-2 (EDI-2) (Paul & Thiel, 2005), The Eating Attitudes Test (EAT-26) (Garner et al., 1982), The Dutch Eating Behaviour Questionnaire - Restraint (DEBQ-R) (van Strien, T. et al., 1986), Self-report version of the Structured Interview for Anorexic and Bulimic Syndromes (SIAB-S) (Fichter & Quadflieg, 1999). To analyze body image, we used Body Shape Questionnaire - Short Version (BSQ-8c) (Pook et al., 2008; Tuschen-Caffer et al., 2005), Body Image Avoidance Questionnaire - Short Version (BIAQ) (Legenbauer et al., 2007), Socio-cultural Attitudes Towards Appearance Scale (SATAQ-3) (Thompson et al., 2004).

### Protocols used

Five different protocols were used in the studies found: (I) Protocol that uses practices based on mindfulness and acceptance of body image, with exercises adapted from Mindfulness-Based Cognitive Therapy (MBCT) for depression [14, 20]; (II) Curriculum.b (“Dot be”), based on mindfulness programs for adults and modified for adolescents [6, 17]; (III) Modified Mindfulness-Based Stress Reduction (MBSR) Program for Adolescents [22]; (IV) MaiStage (Mainz School Training of Eating Disorder Prevention) [23]; and (V) The b@Ease Mindfulness App [21].

In-person MBIs took place in a minimum of three meetings and a maximum of nine, all at weekly intervals. The meetings lasted a minimum of 35 min and a maximum of 90 min. Except for the study by Turner [21], the others carried out follow-up at one or two moments, the shortest interval being 1 month after the intervention, and the longest, 12 months.

Five articles reported that MBI practices at home were suggested during interventions [6, 14, 17, 22, 23]. Only two articles cited no reports of adverse effects during interventions [21, 22]. The others did not address the issue.

### Effects of MBIs on adolescent eating behavior

Of the six articles selected, three [14, 21, 22] proposed to investigate the effects of MBIs on eating behavior, of which two identified related factors [14, 21].

Atkinson and Wade [14], studying preventive effects of an MBI in female adolescents, observed significant reductions in dietary restriction as well as eating disorder symptoms, even 6 months after the intervention, when practices were guided by a trained facilitator. This study analyzed effects of MBI on both eating behavior and body image.

In 2017, Turner e Hingle [21] developed “The b@Ease Mindfulness App”, a cell phone application for adolescents, identifying reports of greater awareness of eating behaviors. Mindfulness practice through a cell phone application has been shown to have the potential to improve eating behaviors, encourage physical activity, facilitate sleep and improve general well-being, thus contributing to awareness of weight-related behaviors.

Salmoirago-Blotcher et al. [22] did not observe differences in eating habits immediately after the interventions or in the 6-month follow-up when applying an MBI.

### Effects of MBIs on adolescent body image

Concerning the effects of an MBI on body image, Atkinson and Wade [14] observed significant reductions in concerns about weight and shape as well as in internalization of a thin ideal, psychosocial impairment and sociocultural pressures.

Buerger et al. [23] proposed to investigate the effects of an intervention that uses mindfulness in its protocol on factors related to body image. The authors suggested that such interventions may be suitable for preventing and protecting adolescents from body dissatisfaction. The effects of the MaiStep protocol demonstrated a relationship with the reduction of negative affect. The authors found positive results in the avoidance of thoughts and behaviors related to body image in the total sample and a reduction in body dissatisfaction post-intervention in the group of adolescents who did not screen positive for eating disorders; however, this last result did not persist in follow-up. It is worth mentioning that the protocol used in the aforementioned study is based not only on mindfulness. Experience-based approaches, group discussions and role-playing are also part of the teaching methods used. Thus, the positive effects on body image may not be entirely related to mindfulness.

Johnson et al. [17] assessed concerns about weight and shape, as well as factors such as anxiety and depression, well-being, mindfulness status, emotional dysregulation and self-compassion, using the.b (“Dot be”) protocol. Despite all the effort, the results found were not significant, either immediately after the intervention or at the three-month follow-up. In 2017, the same authors tried to adjust the gaps in the previous study, but still no significant results were found either in the immediate post-intervention or in the six or twelve month follow-up [6].

## Discussion

MBIs have been extensively studied in adult and clinical populations; however, there is a lack of application of these interventions in universal programs for young people. The main objective of this scoping review was to explore the benefits of MBIs and their relationships with body image and eating behavior in non-clinical adolescent populations. The results were the scarcity of the topic in the literature and the positive effects of MBIs on body image and eating behavior. Of the six studies selected, all were published in the last nine years, which demonstrates the relevance of the topic.

Most studies took place in a school setting, and this can be considered a positive factor, given the importance of universal programs such as those applied in schools in prevention and/or identification of adolescents with subclinical or already established eating disorders who do not have access to the necessary interventions individually or who do not seek help [14].

As for the sex of the populations, the majority worked with both sexes, and this is important since men, like women, suffer social pressure to adapt their bodies to hegemonic beauty standards and masculinity ideals, which can be a risk factor for eating behaviors and physical exercise practices that compromise health [24]. Despite this, literature on the investigation of eating disorders in men is still scarce.

The ages covered by the studies found in this review varied, making it possible to analyze the effects of interventions that used mindfulness in different age groups. Of the six studies analyzed, three found some positive relationship between interventions that used mindfulness, eating behavior and/or body image. Of these three studies that demonstrated some effect, all adopted the smallest time intervals for the duration of interventions, such as three [14, 20], five [23] or six [21] weeks, and two covered the age range of 14–18 years [14, 20, 21]. Johnson et al. [6]. mention that older adolescents present important differences in neurocognitive maturity, which may contribute to the effectiveness of MBIs, and this age group may respond better. However, older students may be more resistant to participating in MBI programs because they interfere with their study time [14]. Possibly, interventions that last fewer weeks are more attractive to this audience, and this makes an exception to what Krebs et al.. propose [25], who, studying MBIs in children and adolescents aged 9–12 years, observed that interventions with shorter and more frequent sessions can be positive in maintaining participants’ involvement and attention.

In 85% of studies found, there was follow-up. Atkinson and Wade [14] highlighted the need for this follow-up, as there is an increase in the size of the effects throughout practice, i.e., mindfulness takes time to confer benefits. Despite carrying out follow-up after six months, the authors suggested longer follow-ups to monitor the potential of mindfulness in producing these benefits, including the importance of qualitative analyzes for discussing strong topics that quantitative data were unable to identify.

Salmoirago-Blotcher et al. [22], when using an adaptation of the traditional Mindfulness-Based Stress Reduction (MBSR) program modified to meet adolescents’ needs during health education classes, they did not observe statistically significant differences in eating habits. The authors analyzed the effects before, immediately after the interventions and after six months, and one of the limitations cited was the collection of only one 24-hour recall.

Using digital technologies such as an app is an approach that can attract adolescents. “The b@Ease Mindfulness App” was created for adolescents, aiming to encourage improvements in diet, physical exercise and sleep, in a flexible, light and fun manner. The interventions were successfully used to improve mindfulness with a focus on nutrition and physical exercise [21]. Supporting these findings, Zhang, Q., O’Connor, D.B. and Hugh-Jones, S [26]., studying the application of an MBI via a smartphone to Chinese adolescents living with excess weight, observed significant positive changes in eating behavior as well as the feasibility and effectiveness of the methodology. Using Information and Communication Technology (ICT) has the advantage of conveying information in a more interactive and personalized manner, having greater acceptance among young people [27]. The strategy of “entering the world of adolescents” is necessary, and in order to directly reach this audience, there is a need to understand that a large part of their world takes place virtually. Taking advantage of this opportunity is a way to even bring more quality to screen time.

Of the six articles selected by this scoping review, the study by Atkinson and Wade [14] was the only one that observed effects of MBIs on both eating behavior and body image. Despite finding no relationship between mindfulness and negative affect, aiming to improve body image, the authors identified positive impacts of MBIs on concerns about weight and shape, sociocultural pressures as well as dietary restrictions, reinforcing that important findings such as these can act in eating disorder prevention in the adolescent population.

A secondary data analysis described by Atkinson and Wade [14] conducted by Osborne et al. [20] demonstrated that MBIs reduced concerns about weight and body shape as well as negative affect, through improved emotional regulation. The authors cited that the effects of mindfulness may have a positive impact on factors such as diet and prevention of disordered eating behaviors.

Observational evidence suggests that MBIs can act to reduce impulsivity– a common characteristic of adolescents– and thus improve issues related to eating [22, 28, 29]. MBIs help identify dysfunctional thought patterns and help individuals get rid of them, being proven effective in addressing body concerns and improving awareness of different parts of the body [30]. Subjects with greater mindfulness have better emotional regulation, which can reduce negative affect, concerns about weight and body shape, increasing hope in preventing eating disorders and their consequences [20, 23].

Johnson et al. [6, 17], using the.b (“Dot be”) program, adapted for Australian adolescents, which is based on MBCT/MBSR adult programs, they did not find any significant results. The authors cited the existence of a scientific gap regarding the best mindfulness protocol for each period of adolescence, justifying the incomplete neurocognitive development at this stage.

Given the heterogeneity of articles published on mindfulness in adolescents, Krebs et al. [25] suggested that units or contents of programs that have proven to be effective in preventing eating disorders be compiled into a single program, thus avoiding wasting time creating other protocols.

Among the studies analyzed in this scoping review, some authors cited difficulties in generalizing results due to the populations studied being too specific, small samples, large intervention groups -with difficulties in providing support to all participants, doubts regarding the need for an instructor with training in mindfulness as well as their expertise in practice with adolescents.

This scoping review identified a variability in sample sizes. A small sample size can be seen as a limitation of this study; however, for conducting interventions, this factor can be seen as beneficial, as it provides more attention and individual instruction to participants [25]. We also identified in the selected studies a heterogeneity in the instruments used to assess eating behavior and body image as well as MBI protocols, with different educational resources, duration and frequency of meetings, presence of follow-up and training of facilitators.

Some scientific gaps were identified, among which the definition of the ideal content and dosage for MBIs aimed at adolescents (frequency, duration of each meeting, application of follow-up) stand out. Johnson et al. [6] highlighted that the identification of mediators and moderators can contribute important information to determine the ideal dose for this audience. Buerger et al. [23] emphasized the need to establish ideal doses in universal prevention programs, in addition to suggesting that booster sessions be included to ensure the acquisition of long-term effects, a statement corroborated by other authors [6, 17]. Atkinson and Wade [14] suggested that a 6-month follow-up is short when the aim is to prevent problems related to body image and eating behavior. The authors suggested that longer follow-ups may be beneficial for maintaining the observed effects. The suggestions raised are in line with something seen in this review and previously mentioned: the three studies that demonstrated the effects of MBIs on body image and eating behavior in adolescents adopted the shortest time intervals for the duration of the interventions: three [14], five [23] or six [21] weeks.

Conducting research that adopts mixed methods can offer important contributions to filling many of these gaps. Qualitative analyses, in particular, allow for a deeper exploration of adolescents’ reflections on their impressions of the encounters, the topic, and the insights (reflections, feelings, thoughts, etc.) perceived during the practices [30]. In this regard, listening to participants’ opinions on aspects such as the content covered, the duration and number of meetings can guide the scientific community towards a more homogeneous and well-founded understanding.

## Conclusion

From the research carried out for this scoping review, it was possible to identify a lack of investigations that analyze the effect of MBIs on eating behavior and body image in adolescents.

Through the studies investigated, we identified that MBIs and interventions that use mindfulness as part of their program appear to positively influence eating behavior (reducing dietary restrictions and symptoms of eating disorders) and body image acceptance (through improved emotional regulation and, consequently, negative affect). However, further research is needed to establish a more robust statement about the efficacy and mechanisms underlying these effects. Heterogeneity among the methodologies used was observed: variations in sample sizes, age groups and gender of participants, and protocols used, which prevents generalization of results to adolescents and corroborates the need to create an ideal protocol for practicing mindfulness with this population.

Analyses using mixed methods can investigate participants’ perceptions about the most effective approach to implementing interventions, considering aspects such as duration and number of meetings. Such contributions can therefore provide valuable insights for improving future research involving MBIs, body image and eating behavior of adolescents.

## Electronic supplementary material

Below is the link to the electronic supplementary material.

**Supplementary Material 1**: **Additional file 1**: Chart 3– Studies about MBIs and relationships with body image and/or eating behavior in adolescents. Recife, 2024.

## Data Availability

No datasets were generated or analysed during the current study.

## Abbreviations

MBI: Mindfulness-based intervention
MBIs: Mindfulness-based interventions
