# Supplementary material for: Mindfulness-based interventions and their relationships with body image and eating behavior in adolescents: a scoping review
Source: J Eat Disord. 2025 May 1;13:77. doi: 10.1186/s40337-025-01238-6 (PMC12044757; doi:10.1186/s40337-025-01238-6)
Supplement: Supplementary file 1 — Supplementary Material 1: Additional file 1: Chart 3– Studies about MBIs and relationships with body image and/or eating behavior in adolescents. Recife, 2024. [file 40337_2025_1238_MOESM1_ESM.docx]

Chart 3 – Publications about MBIs and relationships with body image and/or eating behavior in adolescents. Recife, 2024

| **DATA ANALYZED** | **AUTHORS, YEAR OF PUBLICATION, COUNTRY OF STUDY**  **STUDY DESIGN**  **SAMPLE SIZE AND SEX**  **AGE OF PARTICIPANTS** | **OBJECTIVES** | **NUMBER OF MEETINGS/DURATION OF EACH MEETING**  **WAS THERE PRE-, POST-FOLLOW-UP ASSESSMENT?** | **WHO GUIDED THE PRACTICES?** | **MAIN RESULTS** | **CONCLUSION** |
| --- | --- | --- | --- | --- | --- | --- |
| **MBI AND EATING BEHAVIOR** | Salmoirago-Blotcher, E; Druker, S; Frisard, C; Dunsiger, SI; Crawford, S; Meleo-Meyer, F; Bock, B; Pbert, L., 2018, USA  Randomized and mixed controlled trial (Quantitative and qualitative)  n=53 girls and boys  Age 12-15 years (average 14.5 years) | Determine the feasibility and acceptability of integrating mindfulness into schools and explore its possible effects on healthy behaviors. | Health education (all): 4 days/week, for 2 consecutive weeks.  Attention control group and mindfulness group: 8 meetings of 45 minutes, with weekly breaks.  Baseline, post-intervention and 6-month follow-up. | Health education: professors.  Mindfulness training: instructor graduated from the Center for Mindfulness professional training program, with experience teaching mindfulness with adolescents as well as current personal mindfulness practice. | No significant differences were observed in eating behaviors, however, mindfulness improved physical activity at the end of the interventions and after 6 months in males and the most active. | Mindfulness in health education classes was feasible and acceptable and showed promising effects on physical exercise behaviors. |
|  | Turner, T; Hingle, M., 2017, USA  Single-arm pilot  n=15 girls and boys  Age 14-18 years (mean 16.5 years, standard deviation +/-1.4) | Assess the feasibility, acceptability, and usefulness of a mindfulness-based mobile application. | 6 weeks/once a day  Videos from 2-15’  (5.5 hours of mindfulness-based content and 3 hours of guided practice over 42 days) | Mindfulness-based videos in the app. | High adherence to the app, especially during physically active practices. Adolescents reported greater awareness of eating behaviors. The app increased participants’ ability to initiate and maintain mindfulness by 2.5 times. | The mindfulness-based mobile app has the potential to improve awareness of weight-related behaviors. |
| **MBI, EATING BEHAVIOR AND BODY IMAGE** | Atkinson,MJ; Wade,TD., 2015, Australia  Randomized and mixed controlled trial (Quantitative and qualitative)  n=347 girls  Age 14–18 years (mean 15.7 years, standard deviation +/- 0.77 | Assess the feasibility, acceptability, and effectiveness of a new MBI to reduce the risk of eating disorders among female adolescents with both a trained and non-expert facilitator. | 3 meetings at weekly intervals/Does not mention the duration of each meeting.  Pre, post-intervention, 1- and 6-month follow-up. | A professor and three graduate students in psychology (trained by her). | Group that had a trained facilitator demonstrated significant reductions in preoccupation with weight and shape, dietary restriction, internalization of a thin ideal, eating disorder symptoms, and psychosocial impairment relative to control at the 6-month follow-up, but without improvements in negative affect. | Mindfulness can be effective in preventing eating disorders. These effects are not immediate, they become more visible over time and seem to depend on a certain level of knowledge and experience on the part of facilitators. |
| **MBI AND BODY IMAGE** | Buerger A, Ernst V, Wolter V, Huss M, Kaess M, Hammerle F., 2019, Germany  Randomized and mixed controlled trial (Quantitative and qualitative)  n=1,654 girls and boys  Age 12-15 years (mean 13.35 years, standard deviation +/- 0.76) | Develop and assess a universal eating disorder prevention program for adolescents of both sexes. | 5 90-minute meetings, with weekly breaks  Data collection at baseline, post-intervention and 12-month follow-up. | Intervention group 1: psychologists;  Intervention group 2: trained professors;  Active control group: professors. | Significant decline in avoidance of body image-related thoughts and behaviors and an improvement in "interoceptive awareness" post-intervention and after 12 months. In the healthy subsample (which did not score risk for eating disorders), a significant decrease was found in the “drive for thinness” and in the Body Shape Questionnaire post-intervention, but not after 12 months. | The MaiStep program improves interoceptive awareness and reduces body image avoidance, and can be used for the universal prevention of eating disorders in children under 15 years of age. |
|  | Johnson, C; Burke, C; Brinkman, S; Wade,T., 2017, Australia  Cluster randomized controlled trial  n=555 girls and boys  Mean age 13.44 years (standard deviation +/- 0.33) | Conduct a rigorous assessment of the .b (“Dot be”) mindfulness curriculum with or without parental involvement compared to a control condition. | 9 meetings of 40–60 minutes, with weekly breaks  For students allocated to the mindfulness group with parental involvement, parents participated electronically.  Post-intervention, 6-and 12-month follow-up. | External facilitator with ten years of personal practice, trained for adults, with .b certification. | Although this study attempted to fill gaps in the previous study (below), there was no difference in results between any of the groups: neither post-intervention nor follow-up. | More research is needed to identify the optimal age, content, and duration of mindfulness programs for adolescents in universal prevention settings. |
|  | Johnson C, Burke C, Brinkman S, Wade T., 2016, Australia  Cluster randomized controlled trial  n=308 girls and boys  Mean age 13.63 years (standard deviation +/- 0.43) | Assess whether the promising effects of IBMs could be replicated in schools; investigate anxiety, depression, well-being, weight and shape concerns in order to evaluate this intervention as a transdiagnostic prevention program; assess whether any benefits were moderated by increased adherence to home practice. | 8 meetings of 35 to 60 minutes, with weekly breaks  Baseline, post-intervention and 3-month follow-up | The first author, a mindfulness practitioner with ten years of personal practice, with .b curriculum certification, and adult facilitator training, prior to the start of the study, she led a small pilot group of community youth with the .b program to establish familiarity with the curriculum. | No significant improvements were found in any of the variables immediately after the intervention as well as after three months. | Further research, including investigation of mediators and moderators in experimental designs, is needed to identify active ingredients and optimal dose in MBIx in school settings. |
